# Gestational gigantomastia with pseudoangiomatous stromal hyperplasia – a case report of rare entities

**DOI:** 10.1093/jscr/rjae835

**Published:** 2025-01-07

**Authors:** Sophia Moore, Carlos Neblett, Kenneth Appiah, Rory Thompson

**Affiliations:** Division of Plastic & Reconstructive Surgery, Department of Surgery, Kingston Public Hospital, North Street, Kingston, Jamaica; Division of Plastic & Reconstructive Surgery, Department of Surgery, Kingston Public Hospital, North Street, Kingston, Jamaica; Division of Plastic & Reconstructive Surgery, Department of Surgery, Kingston Public Hospital, North Street, Kingston, Jamaica; Department of Pathology, University Hospital of the West Indies, Kingston, Jamaica

## Abstract

Gestational gigantomastia (GG) is a rare and severe clinical complication of pregnancy. It is characterized by dramatic and uncontrolled growth of the breasts, often leading to physical discomfort, psychological distress and significant surgical complications. Its pathophysiology is poorly understood; management options include conservative pharmacological and surgical interventions. Pseudoangiomatous stromal hyperplasia of the breast is a very rare, incidental, and histological diagnosis seen predominantly in women aged 30–40 years old, with the management generally involving surgical excision. The authors herein discuss an unusual case of bilateral GG complicated by pseudoangiomatous stromal hyperplasia in a premenopausal Caribbean woman, which is the second reported case in this population, with the first reported by one of our authors.

## Introduction

Gestational gigantomastia (GG) is a rare condition that affects 1 in 28 000 to 100 000 pregnancies [1]. It’s characterized by diffuse, extreme unilateral, or bilateral breast enlargement during the first or early second trimester, as described by Palmuth in 1968 [2, 3]. Pseudoangiomatous stromal hyperplasia (PASH) of the breast—described in 1986 by Vuitch *et al.*—comprises mammary stromal proliferations with complex anastomosing channels lined with slit-like spindle cells which mimic blood vessels [4]. PASH, also being rare with no clearly defined prevalence, is usually an incidental finding on biopsy [5]. Our case of GG with PASH is the second of its kind in our region that has been documented.

## Case report

A 28-year-old woman with no chronic illnesses presented to the plastic surgery outpatient department with a history of bilateral macromastia, after Wise pattern inferior pedicle reduction mammoplasties 2 years prior, complaining of acute rapid breast enlargement. The breasts nearly doubled in size over 4 weeks with associated pain, skin changes, and drainage. Historically, the larche was at 10 years of age, menarche at age 13, with rapid breast growth around 16 years of age—though hormonal panel at that time had no abnormalities—associated with large breast masses which were histologically reported as fibroadenomas. There was hormonal contraceptive usage 2–3 years before undergoing reduction mammoplasty at age 26 years. However, at the time of presentation she was not on hormonal contraception and her last menses was 3 weeks prior. No family history of breast cancer or macromastia.

**Figure 1:**
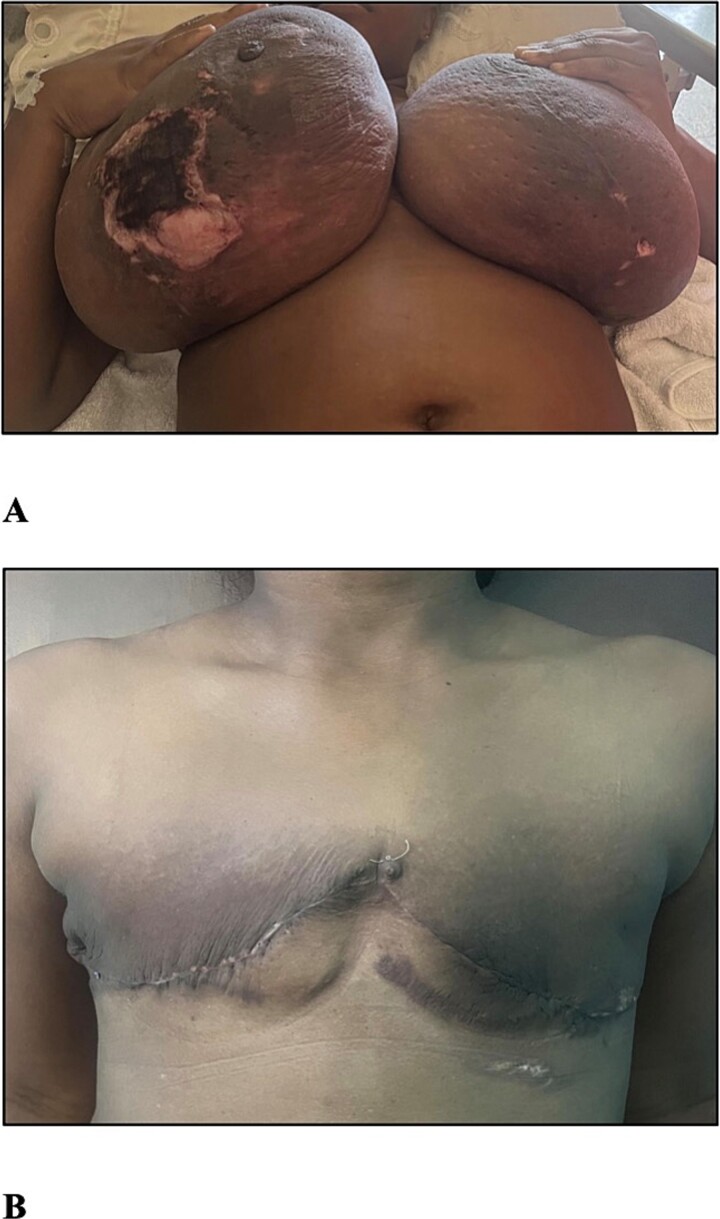
(A) The photograph shows bilateral gestational gigantomastia R > L with skin changes. (B) The photograph shows the patient 1 month post bilateral simple mastectomies.

Examination revealed a woman with an elevated body mass index (BMI) of 34.03 kg/m^2^, bilaterally enlarged tender breasts with erythema right greater than the left, tissue necrosis of the inferior poles, right nipple-areola complex necrosis, and serous drainage. There was no associated palpable lymphadenopathy (Fig. 1A).

Ultrasound evaluation demonstrated diffuse inflammatory changes to the breast parenchyma with no discrete collections, multiple round/ovoid hypointense foci with well-circumscribed margins, and an associated enlarged left axillary lymph node.

The client was subsequently managed in hospital for pain control and local wound care of the right breast as she was overwhelmed, which prompted a request for surgical intervention. After a multidisciplinary team meeting a decision was made for staged total mastectomies. Four days after presentation, a right total mastectomy was performed with the specimen weighing 2.4 kg.

On post-operative Day 2, the left breast findings progressed with further skin necrosis and drainage along with associated fever, prompting a left total mastectomy, with a specimen weighing 2.1 kg.

**Figure 2:**
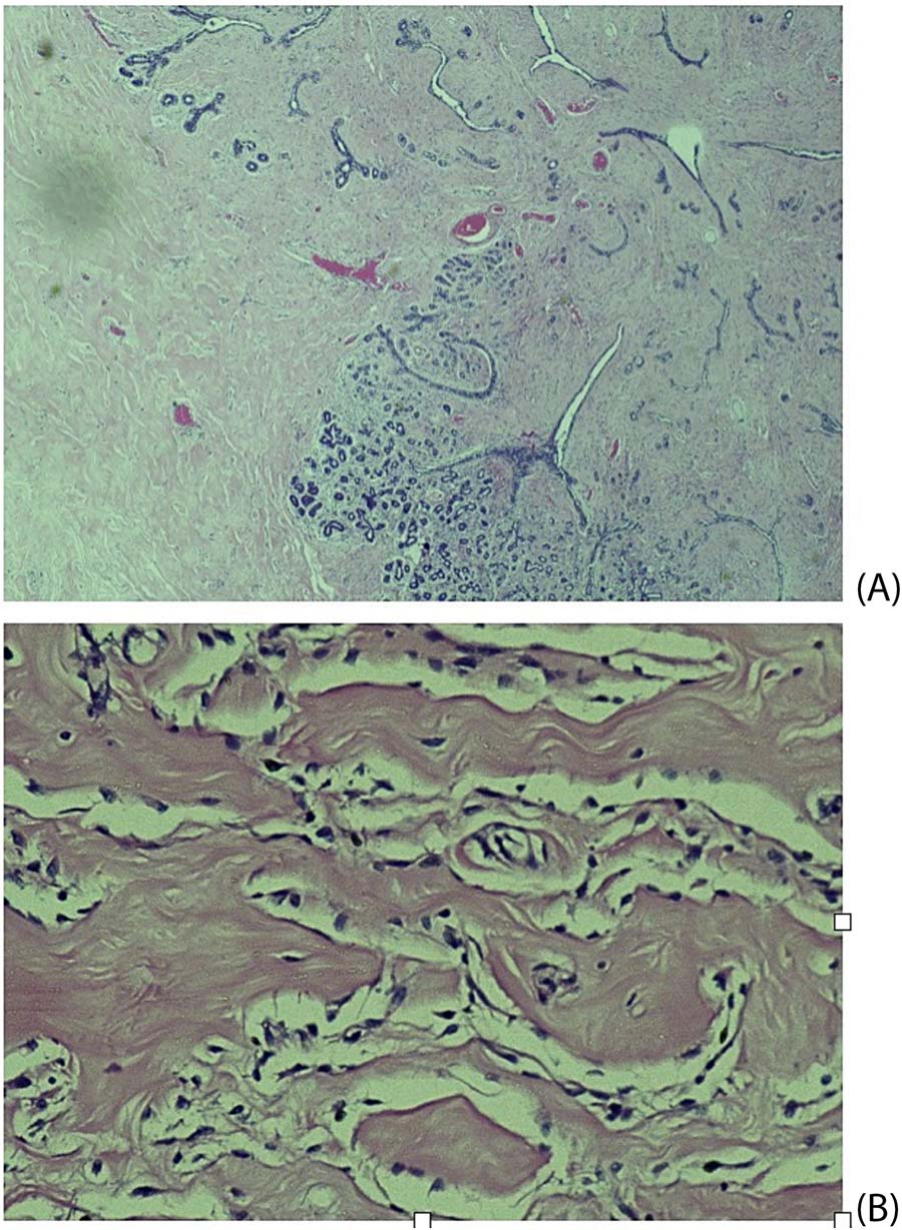
Histological slides showing: (A) Fibroblastic proliferation with cleft like spaces within and adjacent to lobular sclerosing hyperplasia (×20). (B) Cleft-like spaces lined by benign flattened stromal myofibroblasts (×200).

**Figure 3:**
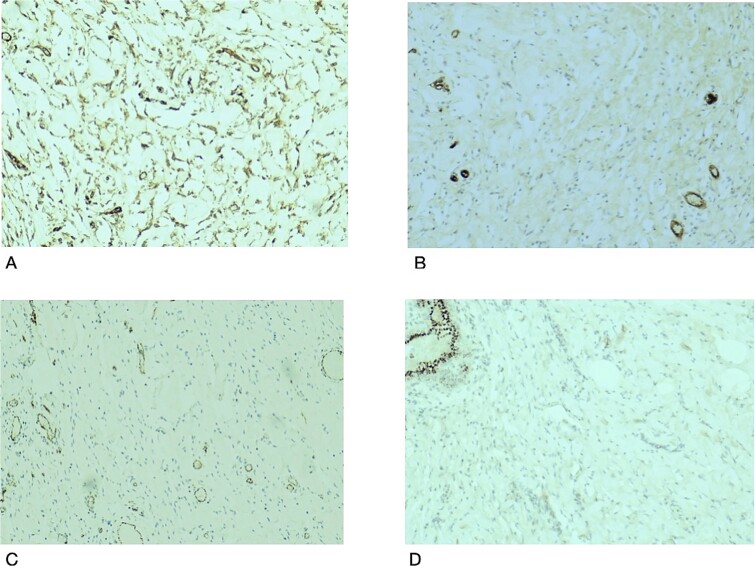
Histological slides showing immunohistochemistry stain expression: (A) Myofibroblasts and endothelial cells showing immune expression for CD34 (×100). (B) CD31 immunostain highlights endothelial cells in blood vessels with no immunoexpression in the stromal myofibroblasts (×100). (C) Factor VIII immunostain of endothelial cells in the blood vessels with no immunoexpression in the stromal myofibroblasts (×100). (D) PR immunostain showing wild type staining in benign breast ducts with no immunoexpression in the stromal myofibroblasts (×100).

Histopathological examination revealed breast tissue exhibiting expansion of the inter- and intralobular spaces by a banal proliferation of stromal myofibroblasts with retraction of the fibrocollagenized stroma, resulting in the formation of cleft-like spaces. The proliferation exhibited a nodular growth pattern, extending into areas of lobular sclerosing hyperplasia (fibroadenomatoid mastopathy). The stroma was paucicellular with foci of cellular fascicular growth (Fig. 2). This overall picture is that of fibroadenoma with pseudoangiomatous stromal hyperplasia. Immunohistochemistry evaluation showed myofibroblasts strong immunoexpression for vimentin and CD34, while CD31 and factor VIII demonstrated no immunoreactivity. The glandular epithelium showed wild-type expression of both oestrogen and progesterone receptors (Fig. 3). This was further suggestive of PASH.

Hormone studies showed elevated free triiodothyronine 4.35 pg/ml (N 2.37–3.91) and prolactin levels 80.14 ng/ml (N 4.79–23.30). The prolactin levels were <10 times the upper limit of normal and suggestive of no pituitary disease - no brain imaging was indicated. There were decreased levels of free thyroxine 0.69 ng/dl (N 0.70–1.48) and luteinizing hormone 0.1 IU/L (normal ranges varies by cycle) with normal levels of thyroid-stimulating hormone 0.46 mI U/ml (N 0.28–3.45) and follicle-stimulating hormone 0.3 IU/L (normal ranges varies by cycle). The lab had no reagent for estradiol.

The post-operative period was uneventful except for an ultrasound diagnosed live intrauterine gestation of 30 weeks after three consecutive missed menses with negative urinary beta-human chorionic gonadotropin (hCG) testing. She was counselled on delayed breast reconstruction, but being symptom-free was not interested and she was satisfied with her outcome (Fig. 1B). The diagnosis was amended to that of GG presenting in the second trimester with associated PASH.

## Discussion

GG is defined as breast weight exceeding or equal to 1.5/2 kg per side or >3% of total body weight in pregnant women [6, 7]. Risk factors include pre-pregnancy macromastia, high pre-pregnancy BMI, gestational diabetes mellitus, and multiparity. These are associated with increased hormonal fluctuations and gestational weight gain, which can contribute to GG. The client, in our index case, had pre-pregnancy mammary hypertrophy, high BMI and elevated prolactin levels.

GG causes physical discomfort with skin changes, emotional distress, and challenges with mobility, ultimately affecting the individual's overall well-being [8], as seen in our index case. Several modalities for managing GG include conservative, pharmacological, and surgical approaches. Conservative management includes supportive garments, physical therapy, and counselling but has limited success rates. A review of the published literature noted only 3 in 50 cases had spontaneous resolution [2]. Pharmacological management can have success rates ranging from 30% to 80% in halting the progression of breast growth and alleviating symptoms, but with limited value in reducing breast volume [9]. Surgery, the mainstay of management includes reduction mammoplasty and mastectomy, is successful in providing relief in >90% of cases [10, 11], as was seen in our patient.

Our case was interesting because it demonstrated PASH, the fascicular/proliferative subtype, defined by the proliferation of the stromal myofibroblast and fibroblasts [12]. Typical PASH is progesterone, estrogen and androgen receptor-positive. The stromal cells express CD34, vimentin, BCL2, CD99, and alpha-smooth muscle actin positivity and are negative for CD 31 and FVIII [13], as reflected in our index case with CD34 and vimentin positivity. PASH is not a malignant precursor; the accepted management is wide local excision in symptomatic and enlarging lesions. Small lesions are observed while, tamoxifen use has been debated [12, 14, 15].

In summary, our client had symptomatic gigantomastia with PASH and a delayed diagnosis of pregnancy. She was managed with mastectomies, and reports improved quality of life.
